# The Cumulative Effect of Transient Synchrony States on Motor Performance in Parkinson's Disease

**DOI:** 10.1523/JNEUROSCI.1975-19.2019

**Published:** 2020-02-12

**Authors:** Gerd Tinkhauser, Flavie Torrecillos, Alek Pogosyan, Abteen Mostofi, Manuel Bange, Petra Fischer, Huiling Tan, Harutomo Hasegawa, Martin Glaser, Muthuraman Muthuraman, Sergiu Groppa, Keyoumars Ashkan, Erlick A. Pereira, Peter Brown

**Affiliations:** ^1^Medical Research Council Brain Network Dynamics Unit at the University of Oxford, OX1 3TH Oxford, United Kingdom,; ^2^Nuffield Department of Clinical Neurosciences, John Radcliffe Hospital, University of Oxford, Oxford OX3 9DU, United Kingdom,; ^3^Department of Neurology, Bern University Hospital and University of Bern, 3010 Bern, Switzerland,; ^4^Neurosciences Research Centre, Molecular and Clinical Sciences Research Institute, St George's, University of London, London SW17 0RE, United Kingdom,; ^5^Movement Disorders and Neurostimulation, Department of Neurology, University Medical Center of the Johannes Gutenberg University Mainz, 55131 Mainz, Germany,; ^6^Department of Neurosurgery,King's College Hospital, King's College London, SE59RS, United Kingdom, and; ^7^Department of Neurosurgery, University Medical Center of the Johannes Gutenberg University Mainz, 55131 Mainz, Germany

**Keywords:** beta oscillations, local field potentials, Parkinson's disease

## Abstract

Bursts of beta frequency band activity in the basal ganglia of patients with Parkinson's disease (PD) are associated with impaired motor performance. Here we test in human adults whether small variations in the timing of movement relative to beta bursts have a critical effect on movement velocity and whether the cumulative effects of multiple beta bursts, both locally and across networks, matter. We recorded local field potentials from the subthalamic nucleus (STN) in 15 PD patients of both genders OFF-medication, during temporary lead externalization after deep brain stimulation surgery. Beta bursts were defined as periods exceeding the 75th percentile amplitude threshold. Subjects performed a visual cued joystick reaching task, with the visual cue being triggered in real time with different temporal relationships to bursts of STN beta activity. The velocity of actions made in response to cues prospectively triggered by STN beta bursts was slower than when responses were not time-locked to recent beta bursts. Importantly, slow movements were those that followed multiple bursts close to each other within a trial. In contrast, small differences in the delay between the last burst and movement onset had no significant impact on velocity. Moreover, when the overlap of bursts between the two STN was high, slowing was more pronounced. Our findings suggest that the cumulative, but recent, history of beta bursting, both locally and across basal ganglia networks, may impact on motor performance.

**SIGNIFICANCE STATEMENT** Bursts of beta frequency band activity in the basal ganglia are associated with slowing of voluntary movement in patients with Parkinson's disease. We show that slow movements are those that follow multiple bursts close to each other and bursts that are coupled across regions. These results suggest that the cumulative, but recent, history of beta bursting, both locally and across basal ganglia networks, impacts on motor performance in this condition. The manipulation of burst dynamics may be a means of selectively improving motor impairment.

## Abstract (precis)

Bursts of beta frequency band activity in the basal ganglia of patients with Parkinson's disease (PD) are associated with impaired motor performance. Here we test in human adults whether small variations in the timing of movement relative to beta bursts have a critical effect on movement velocity and whether the cumulative effects of multiple beta bursts, both locally and across networks, matter.

## Introduction

One of the electrophysiological hallmarks of Parkinson's disease (PD) is exaggerated beta activity (13–35 Hz) in basal ganglia local field potentials (LFPs), which is linked to motor impairment (Brown, 2003). Both the administration of levodopa and the application of continuous high-frequency deep brain stimulation (DBS) suppress this activity in the subthalamic nucleus (STN), with the degree of suppression being positively correlated with clinical motor improvement (Kühn et al., 2006, 2008, 2009; Eusebio et al., 2011; Özkurt et al., 2011; Oswal et al., 2016; Trager et al., 2016). Beta activity also occurs under physiological conditions, where it takes the form of relatively short-lived phasic bursts in basal ganglia-cortical motor circuits (Murthy and Fetz, 1992, 1996; Feingold et al., 2015; Deffains et al., 2018). In contrast, the distribution of beta burst durations is shifted to the right, in favor of longer durations, in untreated PD, and the proportion of long duration beta bursts is correlated with rigidity and bradykinesia (Tinkhauser et al., 2017a,b; Deffains et al., 2018). Both the delivery of beta-triggered adaptive DBS and the administration of levodopa shift the distribution of burst durations toward the left, in association with clinical improvement (Tinkhauser et al., 2017a,b). In the specific case of beta-triggered adaptive DBS, because of the design of the control-algorithm (Little et al., 2013), the effect of stimulation led to the curtailing of beta bursts exceeding ∼500 ms in duration (Tinkhauser et al., 2017b). Thus, it is reasonable to conclude that bursts with at least this duration are associated with motor impairment. But what of bursts shorter than this, which are left untouched by adaptive DBS; could these also contribute to motor impairment in PD? Correlations between the relative prevalence of beta bursts of different duration and clinical motor impairment suggest that bursts with durations less than ∼400 ms might actually be beneficial (Tinkhauser et al., 2017a,b). However, given that the number of bursts with a specific duration was considered as a fraction of all bursts the association of shorter bursts with better clinical state might simply have been secondary to the fact that a greater fraction of shorter bursts necessarily means a smaller fraction of longer bursts. More recently, it has been shown that beta bursts with mean durations of 200–350 ms are also linked to slowing of subsequent voluntary movement, when the latter is objectively measured (Torrecillos et al., 2018; Lofredi et al., 2019).

The precise conditions under which beta bursts impact on movement also remain unclear. For example, are small variations in the delay between bursts and movement important, and do multiple bursts preceding movement have a bigger impact? In addition, it has been demonstrated that beta bursts are coupled across the basal ganglia cortical network (Tinkhauser et al., 2018b), but whether simultaneous bursting across the circuit has any additional impact on subsequent movement is unknown. Here we test whether small variations in the timing of movement relative to beta bursts have a critical effect on movement velocity and whether the cumulative effects of multiple beta bursts, both locally and across networks, matter. To this end we designed an experiment that allowed us to detect beta bursts online, and thereby trigger imperative cues so that we had more precise control over the timing of subsequent voluntary movements.

## Materials and Methods

### 

#### 

##### Subjects and surgery.

We studied 15 patients with advanced PD who underwent bilateral STN-DBS surgery. Their clinical details are summarized in Table 1. Subjects were recruited at three different sites, St. Georges Hospital London (UK), Kings College Hospital London (UK), and Mainz University Hospital (DE). The investigation was approved by the local ethics committees (Mainz University Hospital: 837.208.17 (11042); UK centers: IRAS 46576) and all subjects gave their written informed consent. Depending on center-specific DBS surgical approaches, electrode implantation was either guided by imaging alone (St. Georges Hospital and Kings College Hospital) or by additional intra-operative micro-recordings (Mainz University Hospital). The implanted leads were either the 3389 DBS lead (Medtronic) with four platinum-iridium cylindrical surfaces or Vercise Cartesia Directional Lead (Boston Scientific) with three segmented contacts on Levels 2 and 3. DBS leads were temporarily externalized for 3–6 d.

**Table 1.** Clinical details

Sub	Gender, M/F	Age, y	Disease Duration, y	Pre-Op UPDRS-III, OFF	Pre-Op UPDRS-III, ON	Predominant symptoms	Time Extern, d	DBS lead	Beta Fr peak, online task	Contact pair	Hand	Site
1	M	61	16	50	30	Akinetic-rigid	3	Bost	25	L12	R	St. Georges London
2	M	59	6	48	14	Akinetic-rigid	5	Medt	21	L23	R	St. Georges London
3	M	65	15	77	27	Akinetic-rigid, tremor	5	Bost	18	L01	R	St. Georges London
4	M	48	17	71	37	Tremor	3	Bost	14	R12	R	St. Georges London
5	M	54	7	38	24	Tremor	5	Bost	23	R12	R	St. Georges London
6	M	56	16	51	19	Akinetic-rigid, tremor	4	Medt	19	L12	R	St. Georges London
7	M	66	15	57	34	Akinetic-rigid, tremor	4	Medt	15	L12	R	St. Georges London
8	F	66	10	53	30	Akinetic rigid	4	Bost	15	L01	R	St. Georges London
9	M	61	10	31	19	Akinetic-rigid, tremor	3	Medt	15	L01	R	Mainz, University Hospital
10	F	67	13	18	15	Akinetic-rigid, tremor	3	Medt	19	L23	R	Mainz, University Hospital
11	M	77	7	35	29	Akin-rigid	3	Medt	12	L23	R	Mainz, University Hospital
12	M	65	10	37	9	Akinetic-rigid, tremor	6	Medt	18	L23	R	Kings College London
13	F	70	20	54	19	Akinetic-rigid, tremor	6	Medt	20	L01	R	Kings College London
14	M	69	17	37	18.5	Akinetic-rigid, tremor	6	Medt	23	L23	R	Kings College London
15	M	68	12	40	17	Akinetic-rigid, tremor	6	Medt	25	L12	R	Kings College London
Mean ± SEM (*n*)	**M(12); F(3)**	**63.4 ± 1.9**	**12.7 ± 1.1**	**46.5 ± 3.9**	**22.8 ± 2.1**		**Median 4 (3–6)**		**18.8 ± 1.1**			

15 patients diagnosed with PD were included in this study. The patient's characteristics were as following: 12 males, 3 females; age 63.4 ± 1.9 years; disease duration 12.7 ± 1.1 years; pre-operative UPDRS III off levodopa: 46.5 ± 3.9, on levodopa: 22.8 ± 2.1. The experiment took place 3–6 days (median 4 days) after lead externalisation. The mean beta frequency peak, which was also selected for the online task was at 18.8 ± 1.1 Hz.

Sub, Subjects; M, male; F, female; UPDRS_Unified Parkinson's disease rating scale Part III; Extern, externalization; Bost, Vercise Cartesia Directional Lead (Boston Scientific); Medt, 3389 DBS lead (Medtronic); Fr, frequency; Hand, handedness; R, right; L, left.

##### Signal recording and preprocessing for online triggering of the visual cue.

**Figure 1.**
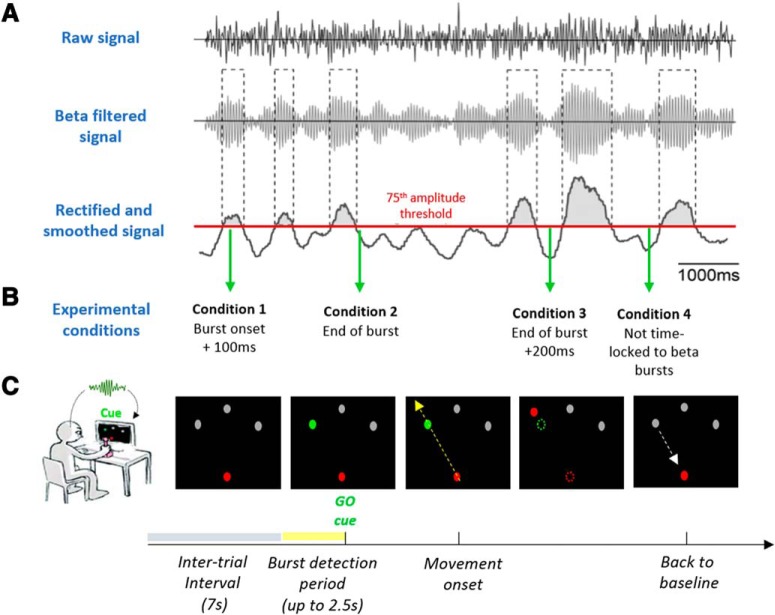
Methods in online experiment. ***A***, The analog LFP signal was filtered around the individual's beta peak frequency (Table 1). The signal was rectified and smoothed (200 ms time constant) to obtain the envelope of the beta signal. To define beta bursts a threshold was set at the 75th percentile of the beta amplitude (red line). The onset of a burst was defined as when the rectified signal crossed the threshold amplitude and the end of the burst defined as when the amplitude fell below threshold. The minimum burst duration was defined as 100 ms. ***B***, The go-cue for the behavioral experiment was triggered according to four conditions. Condition 1–3 were aligned to the beta burst timing. In Condition 1 the go-cue was presented 100 ms after the onset of beta bursts. The waiting period of 100 ms was necessary to capture bursts as previously defined. In Condition 2 the go-cue was presented at the end of the bursts. In Condition 3 the go-cue was presented at the end of the bursts +200 ms. In Condition 4 the go-cue was presented without any fixed temporal relationship to beta bursts (see Materials and Methods). ***C***, The behavioral part of the experiment. The subject controlled the red cursor with a manual joystick and was instructed to perform a ballistic movement in the direction of the go-cue (green target). The intertrial interval was 7 s plus up to a 2.5 s burst detection period necessary to meet one of the randomly assigned Conditions 1–3 (***B***). For each condition a number of 60 trials were aimed for.

Figure 1*A* illustrates the LFP recording and processing steps for the behavioral experiment. All patients were recorded after withdrawal of their dopaminergic medication. Signals were recorded using a TMSi-Porti amplifier (TMS International). The ground electrode was placed on a forearm. LFP signals were amplified, low-pass filtered at 550 Hz, sampled at 2048 Hz and common average referenced. LFPs were off-line reconfigured to give a bipolar contact arrangement between the four electrode levels (directional contacts of one level were connected together to form one “contact”) so that each electrode afforded three bipolar signals for the left (L01, L12, L23) and right (R01, R12, R23) STN. Bipolar montages between adjacent contact pairs were used as they limit the effects of volume conduction from distant sources (Marmor et al., 2017). For subject 15, because of technical reasons, only one bipolar channel was available on the left and right sides. The timing of cue-presentation, the displacement of the response joystick in the *x* and *y* planes and the signal from an accelerometer taped to the dorsum of the active hand were also recorded through the TMSi-Porti amplifier and sampled at 2048 Hz.

Before the experiment started one bipolar channel from either the left or right STN (Table 1) had to be selected for computing the beta bursts online that would trigger the imperative cues. We selected the channel with the highest resting beta activity, or, in the case of similar levels of beta between channels, the channel showing the strongest beta modulation during voluntary hand movements. This step was motivated by evidence linking maximal beta band activity (Chen et al., 2006; Zaidel et al., 2010; Horn et al., 2017) and movement-related beta reactivity (Devos et al., 2006; Tinkhauser et al., 2019) to the dorsal (motor) region of the STN, which also corresponds to the site that offers the most effective deep brain stimulation (Ince et al., 2010; Zaidel et al., 2010; Tinkhauser et al., 2018a). Only one contact pair was selected for each patient and the joystick movement was performed with the contralateral hand.

The signal chosen as trigger was then bandpass-filtered around the individual beta peak (±3 Hz), rectified and smoothed (200 ms time constant). In line with previous work (Tinkhauser et al., 2017a,b, 2018b; Torrecillos et al., 2018) beta bursts were defined by crossings of the 75th percentile amplitude threshold of the beta signal (Fig. 1*A*, red line). The onset of a burst was defined as when the rectified signal crossed the threshold amplitude and the end of the burst defined as when the amplitude fell below threshold. The minimum duration of the threshold crossing to be considered as a burst was set to 100 ms (Tinkhauser et al., 2017b).

##### Behavioral task.

Subjects performed a visually cued joystick reaching task, with the visual cue triggered either by beta bursts in the STN or with no fixed relationship to beta bursts. The task was programmed and synchronized to the LFP recording using in-house developed software written in C++. The paradigm is illustrated in Figure 1, *B* and *C*. Subjects sat comfortably in front of a computer monitor at arm's length. With their right or left hand, i.e., the hand contralateral to the trigger STN channel, they held a joystick which was fixed on a table. The position of the joystick was displayed on the computer monitor as a red circle and localized at the bottom center of the screen when in resting position. At the top of the screen, distributed on a half circle, three potential, equally spaced, circle targets in gray were shown (left side, middle, right side). Once one of the three targets changed color to green (go-cue), subjects were instructed to make a rapid, ballistic movement from the resting position in the direction of the target. The ballistic nature of the response was stressed, and subjects were asked to make a single straight movement that went through the target. To minimize any corrective movements, no visual feedback of the cursor position was provided during the movement. The position of the red cursor was presented at rest, disappeared after movement onset, and reappeared once the movement trajectory went beyond the target. Thereafter subjects could move back to the resting position. The go-cue was triggered according to four different conditions, three of which depended on the timing of beta bursts. At the outset of each trial the likelihood of one or other condition being set was 1 in 4, with the condition type selected randomly. The intertrial interval was 7 s plus up to a 2.5 s period during which our custom-written software searched for a beta-burst configuration that met the preselected condition. The long intertrial interval was chosen to avoid the beta rebound after a movement contaminating the next trial. In Condition 1 the go-cue was presented 100 ms after the onset of a beta burst detected during the burst screening period in the contralateral STN. The waiting period of 100 ms was necessary to avoid including brief threshold crossings <100 ms as bursts. In Condition 2, the go-cue was presented at the end of a burst, when the 75th percentile threshold was again crossed as the beta amplitude ramped down. In Condition 3 the go-cue was presented 200 ms after the end of a burst detected in the screening period, provided no further bursting occurred in this period. In Condition 4 the go-cue was presented without any fixed temporal relationship to beta bursts. This was our reference condition and was primarily achieved by triggering the go-cue at some random time point during the 2.5 s burst screening period, regardless of any particular timing to bursting activity. To these trials were added those in which the software initially set out to have Conditions 1–3, but in which criteria for these conditions were not satisfied. In these trials the go-cue was triggered at the end of the burst screening period. The additional trials in Condition 4 comprised ones in which no burst was detected in the burst screening period (either no burst or threshold surpassed for <100 ms), trials marked for Condition 3 in which a burst was not followed by 200 ms clear of further bursts, and trials in which the beta signal rose above threshold during the burst screening period, but then failed to return below threshold before the end of this period. These trial types still satisfied the overall goal that Condition 4 should represent trials in which go-cues were presented without any systematic time-locking to any beta bursts.

After initial familiarization (10–20 trials) of the task, we aimed to obtain a minimum number of 60 trials per condition. Note, conditions were assigned randomly and all trials subdivided in four to six blocks, with a 5 min break between the blocks. The total experiment duration was 90–120 min.

##### Off-line behavioral analysis.

The data were first visually inspected using Spike2 Software (Cambridge Electronic Design Limited). Trials contaminated by artifacts, by movement during the resting period (detected by the accelerometer on the active hand) or failed trials (e.g., subject did not move) were removed from the dataset. Further analyses were performed off-line using custom-written MATLAB scripts (version R2018b, MathWorks). Motor performance was assessed by the peak velocity (PV) of the joystick movement. We opted for this parameter because of the strong link between bradykinesia and basal ganglia beta bursts (Tinkhauser et al., 2017a,b, Torrecillos et al., 2018; Lofredi et al., 2019). To this end the position of the red joystick cursor was differentiated to calculate the displacement of the joystick over time (movement velocity). Movement onset was defined as the time when the joystick velocity exceeded five-times the SD of the signal at rest. All trials were further visually inspected to check that this onset was correctly defined by this criterion. PV was defined as the maximum velocity in the direction of the target after movement onset. We only considered trials with a reaction time (measured from go-cue to movement onset) <1.5 s, and thereafter also rejected trials in which PV or reaction time exceeded 2.5 times the SD from the mean.

##### Off-line LFP processing and burst determination.

**Figure 2.**
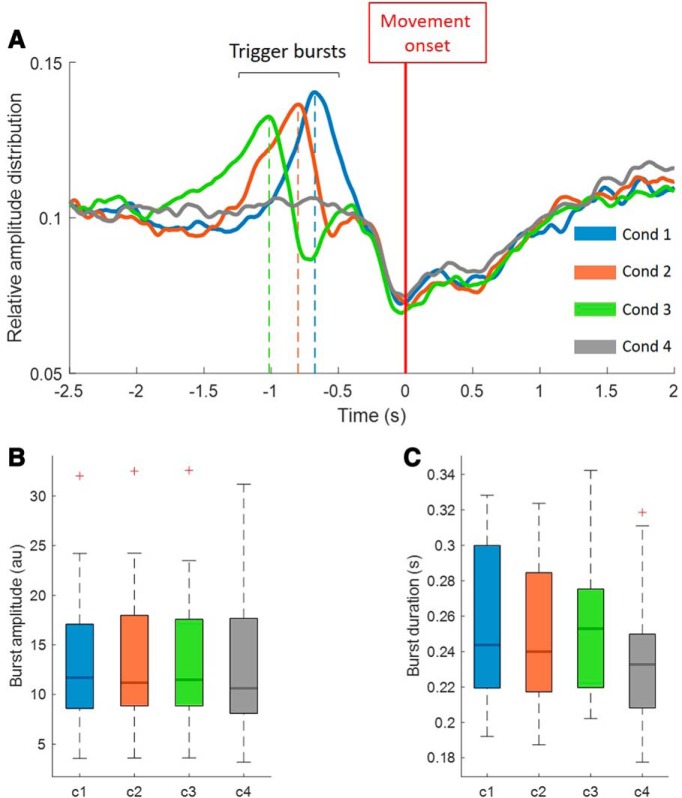
Distribution and characteristics of beta bursts in Condition 1–4. ***A***, The relative averaged beta amplitude for all conditions (1–4) over the period from −2.5 s before the onset of the movement up to 2 s after the movement. The amplitude peaks in Condition 1–3 correspond to the timing of the peak of the trigger bursts (i.e., those triggering the cue) before the onset of the movement (c1= −0.68 s, c2= −0.80 s, c3= −1.02 s). As expected, no such peak can be derived from Condition 4, in which the presentation of the go-cue was not timed with the occurrence of beta bursts. ***B***, ***C***, The averaged maximal burst amplitude and mean burst duration for the bursts detected in Conditions 1–4. Separate RM-ANOVAs gave a significant main effect for the amplitude comparison (*F*_(3,42)_ = 5.21, *p* = 0.021) and for the comparison of burst duration (*F*_(3,42)_ = 4.75, *p* = 0.026). However *post hoc* pairwise comparisons between conditions were not significant after correction for multiple comparisons. Thus, the intrinsic characteristics of beta bursts were considered comparable across the four conditions. Red crosses correspond to values above the 75th percentile.

To explore the trial-by-trial relationship between beta oscillations and motor performance we defined beta bursts again off-line using previously established methods (Tinkhauser et al., 2017a,b, 2018b; Torrecillos et al., 2018). Note, the channel used for further signal processing and analyses was the same bipolar channel in which beta bursts were monitored to trigger cues during the online task (Table 1). LFP signals were resampled to 200 Hz and for each trial decomposed into frequency components with a frequency resolution of 1 Hz using a wavelet transformation (ft_specest_wavelet script in Fieldtrip–Morlet Wavelet: width = 10, gwidth = 5; Donders Institute for Brain, Cognition and Behavior, 2010). All trials were segmented from −3 s up to movement onset, to cover our primary period of interest of −2.5 s to movement onset. The evolution of beta power over time in each trial was computed off-line by averaging over a 6-Hz-wide frequency band centered on the beta peak frequency (Table 1). For each subject we defined a common amplitude threshold, based on the average 75th percentile amplitude of periods from −3 to −1 s to movement onset of trials from the reference Condition 4. We defined the threshold in this condition, because in all other conditions (1–3) beta activity would be artificially elevated because we picked time periods where beta bursts occurred. We considered the period from −3 to −1 s before movement onset to be certain of picking a representative resting period. This common threshold was then applied to redefine bursts in each individual trial from Conditions 1 to 4 off-line. Bursts were defined from threshold crossings as before, and we again excluded bursts with durations shorter than 100 ms to limit the contribution of spontaneous fluctuations in amplitude because of noise. This had to be done again off-line as the smoothing properties of the off-line filter slightly differed from the online filter. Finally, we identified the “trigger-bursts” in Conditions 1–3, i.e., the bursts which triggered the go-cue (Fig. 2*A*). We also identified any additional bursts that followed the trigger-bursts in Conditions 1–3 up to the point of movement and termed these as “continued-bursting”.

##### Extraction of burst dynamics.

We determined burst rate, defined as number of bursts/s occurring before the onset of the movement. If no burst was present during this period, the burst rate for this trial was set to zero. We also considered the effect of the proximity of the last burst in time to movement onset (timing of peak amplitude and end of the bursts relative to the movement onset). Here we only included trials with at least one burst present in the period investigated. Furthermore we investigated the interval between the peak amplitude of successive bursts, where these were multiple within the window of interest. The latter is similar to the burst rate, although not exactly the same as it is also depended on the duration of bursts and only trials with at least two bursts within the window of interested were included. Finally, we considered amplitude modulation in the opposite STN during periods of bursting and non-bursting and determined the “burst overlapping”. As burst overlapping, we considered the percentage time of the entire pre-movement period where bursts overlapped between the hemispheres (Tinkhauser et al., 2018b). Here we only considered trials with at least one burst detected in the reference STN (STN contralateral to the hand used for the joystick movement).

##### Comparisons and statistical analysis.

Statistical analyses were performed using MATLAB (vR2018b; MathWorks). Peak velocities were *z*-transformed and reaction times log-transformed before statistical comparisons. These transformations were performed separately for each subject, on all the trials of the 4 conditions pooled into one group. Conditions 1–3 were either compared separately or as joint group. To test for a systematic difference between the three burst conditions we performed a repeated-measurements (RM)-ANOVA (factors: velocity/reaction time and conditions), with the normality tested before comparison. Assumption of sphericity was checked with Mauchly's test; if violated, *F* and *p* values were reported with Greenhouse–Geisser correction. Comparisons between two groups were performed using a paired nonparametric test (Wilcoxon signed rank test). We turned to condition 4 to study the impact of burst rate, burst interval and burst overlapping on motor performance. To this end, trials were median split according to the parameter of interest. The burst distributions of all conditions before movement onset were calculated using the probability density function provided by MATLAB. To control for multiple comparisons we performed the false discovery rate correction procedure, which controls the expected proportion of falsely rejected hypotheses (Benjamini and Hochberg, 1995). In each box plot presented, the central mark indicates the median and the bottom and top edges of the box indicate the 25th (Q1) and 75th percentiles (Q3), respectively. The whiskers show Q1 − 1.5 × interquartile range (IQR) and Q3 + 1.5 × IQR. Red crosses (+) show outliers beyond this range.

## Results

### Burst characteristics and distribution

In this study we investigated whether the precise time of movement after the onset or offset of a beta burst affects movement velocity and whether the cumulative effects of multiple beta bursts locally or across networks matters. To this end, using the online experiment, as illustrated in Figure 1, we acquired trials in four conditions with different burst timing relationships. The cue in Condition 1 was presented 100 ms after the onset of the trigger-beta burst in each trial, in Condition 2 just at the end of the trigger-burst, in Condition 3 200 ms after the end of the trigger-burst and in Condition 4 the go-cue was presented without any fixed temporal relationship to beta bursts. Across all subjects the mean number of trials (±SEM) finally used for analysis after preprocessing was 58.4 ± 3.7 trials for Condition 1, 56.5 ± 3.5 trials for Condition 2, 56.1 ± 3.1 trials for Condition 3 and 81 ± 8 trials for Condition 4. Note, Condition 4, our reference condition, had a higher number of trials. Conditions 1–3 were associated with distinct beta burst distributions before the onset of the ballistic joystick movement (Fig. 2*A*). The maximums of the burst peaks in averaged data for Conditions 1, 2, and 3 occurred −0.68, −0.80, and −1.02 s before movement onset, respectively. As expected, there was no discrete burst peak in averaged data before movement in Condition 4, where the averaged data continued to be flat over the period of interest. Figure 2*A* therefore demonstrates that the presentation of the go-cue was not time-locked to a beta burst in Condition 4 so that averaged beta was clearly less than that in Conditions 1–3 over the key period of 0.5–1.0 s before movement onset. Note that, in contrast, the characteristics of the detected beta bursts (burst amplitude and burst duration) did not vary between the four conditions (Fig. 2*B*,*C*).

### Triggering off beta bursts slows down movement independent of precise timing

**Figure 3.**
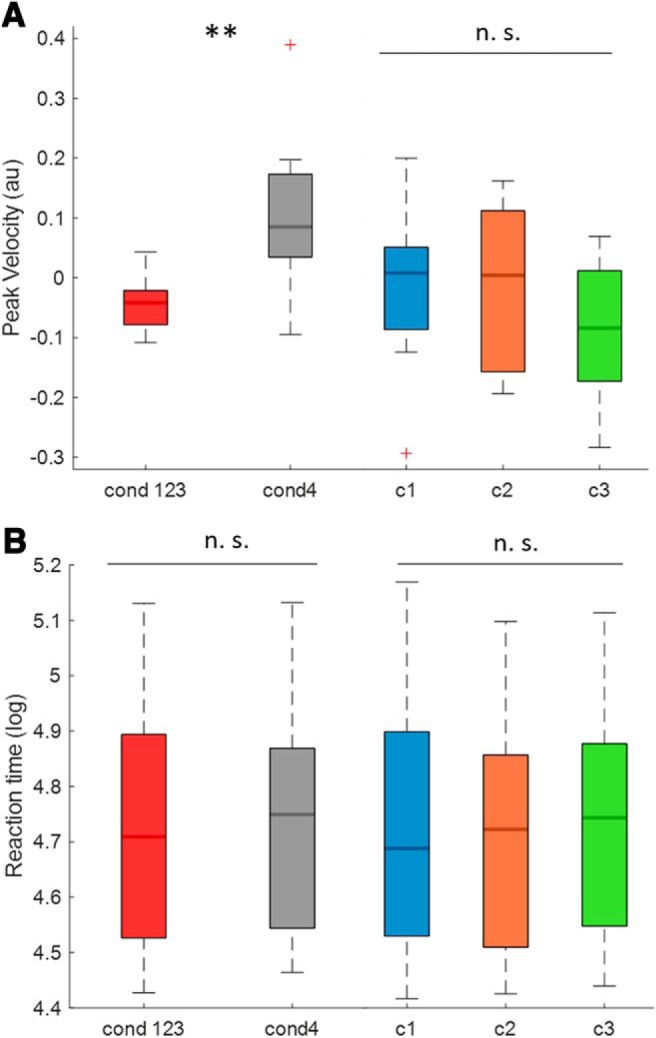
Effect of burst conditions on peak velocity (PV) and reaction time (RT). ***A***, The mean *z*-scored PV of the joint Conditions 1–3 (go-cues time-locked to bursts) and the mean PV of Condition 4 (go-cues not time-locked to bursts). The PV during the burst conditions is significantly slower than in Condition 4 (*n* = 15, *z* = 12, *p* = 0.0043); it also illustrates the PV of Conditions 1–3 individually (burst conditions) across subjects. No significant difference was found between the three burst conditions (RM-ANOVA, no significant main effect, *F*_(2,28)_ = 1.4663, *p* = 0.25). ***B***, The mean log-transformed RT of the joint Conditions 1–3 (burst conditions) and the mean RT of Condition 4 (go-cues not time-locked to bursts). This comparison did not reveal a statistical difference (*n* = 15, *z* = 39, *p* = 0.25); it also illustrates the RT of Conditions 1–3 individually (burst conditions) across subjects. No significant difference was found between the three burst conditions (RM-ANOVA, no significant main effect, *F*_(2,28)_ = 1.693, *p* = 0.20). Red crosses correspond to value above the 75th or below the 25th percentile. **p < 0.01. n.s. = not significant.

Here we test whether the precise time of movement after the onset or offset of a beta burst affects movement velocity. First we determined whether there was a genuine impact of prospectively triggering off beta bursts on motor performance. Accordingly, we collapsed Conditions 1–3 together in to a single group and compared the peak velocity of the ballistic response to that obtained in Condition 4, where go-cues were not time-locked to beta bursts. Figure 3*A* illustrates that if the go-cue is triggered by a beta burst, the peak velocity of the ballistic movement is significantly slower (*n* = 15, *z* = 12, *p* = 0.0043) compared with trials where the go-cues were not time locked to beta bursts (Condition 4). Thus, if a voluntary movement is forced to follow a beta burst within a relatively narrow time window then movement is slowed. Although the trigger-bursts from Conditions 1–3 did not differ with regard to their burst characteristics (Fig. 2*B*), they did vary in their proximity to movement onset as reported above (Fig. 2*A*). So next we asked whether beta bursts peaking at different times before the movement had varying impact on PV. We first compared the individual Conditions 1–3 separately with reference Condition 4 and found that all three conditions showed a trend to slow down more than in the reference condition, but only in Condition 3 did this reach statistical significance [Condition (c)1 vs c4: *n* = 15, *z* = 27, *p* = 0.064; c2 vs c4: *n* = 15, *z* = 23, *p* = 0.053; c3 vs c4: *n* = 15, *z* = 12, *p* = 0.013]. More importantly, we directly compared the PV between Conditions 1–3 (Fig. 3*A*), and found no significant difference (RM-ANOVA, *F*_(2,28)_ = 1.4663, *p* = 0.25). The latter result suggests that the precise timing of beta bursts with peaks within the range of −0.68 to −1.02 s does not have a major impact on motor performance.

We also explored beta burst effects on reaction times. The mean reaction time of subjects was 0.58 s ± 0.03 across the whole task. The comparison of mean reaction times between the collapsed conditions (1–3) with reference Condition 4 (*n* = 15, *z* = 39, *p* = 0.25) showed no difference. Similarly, the comparisons of individual Conditions 1–3 with Condition 4 (c1 vs c4: *n* = 15, *z* = 49, *p* = 0.56; c2 vs c4: *n* = 15, *z* = 24, *p* = 0.12; c3 vs c4: *n* = 15, *z* = 42, *p* = 0.33), as well as comparisons between Conditions 1–3 (RM-ANOVA, *F*_(2,28)_ = 1.693, *p* = 0.20) showed no significant difference (Fig. 3*B*).

Hence for all subsequent analyses we focus on our outcome measure of interest, peak movement velocity.

### Single bursts versus clusters of bursts

**Figure 4.**
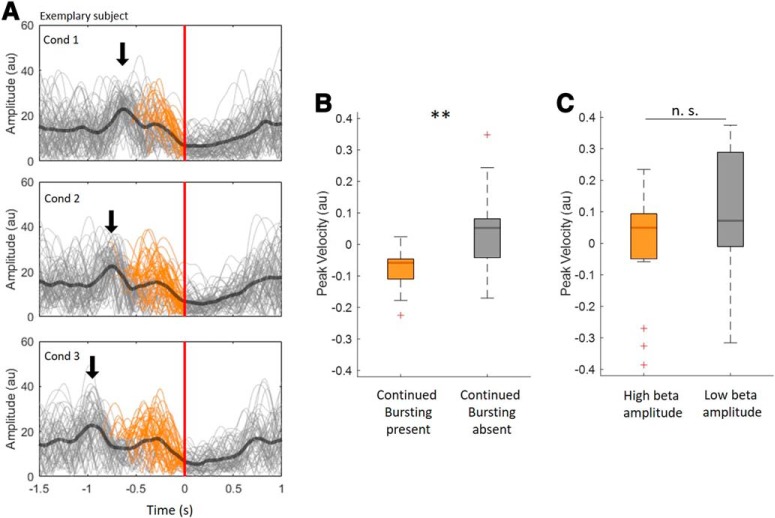
Continued bursting in Condition 1–3 and impact on peak velocity (PV). ***A***, The beta power envelopes of single trials (gray) and the average beta envelope (bold black) for Conditions 1–3 in the representative Subject 7. The dark blue arrow indicates the trigger burst of the three conditions which was used to trigger the go-cue in the online experiment. The trials are aligned to the movement-onset, indicated by the red line at time 0. The orange sections of beta power envelopes indicate trials with additional bursting (continued bursting) after the trigger burst (72.3 ± 2.9% of trials). ***B***, The comparison of PV in trials with continued bursting with those without continued bursting. This reveals that PV in the group with continued bursting was significantly lower than the PV of the remaining trials (*n* = 15, *z* = 15, *p* = 0.008). ***C***, No such difference was found when all trials were median split according to the beta amplitude during the period of continued bursting to give groups of trials with low and with high beta amplitude (*n* = 15, *z* = 36, *p* = 0.188). Red crosses correspond to values above the 75th or below the 25th percentile. **p < 0.01. n.s. = not significant.

Although cues were triggered by a single burst in Conditions 1–3, the interval between triggering and movement execution was such that additional bursts could occur (Fig. 2*A*). Figure 4*A* illustrates all three conditions in an example subject, and shows the trigger bursts and variable subsequent bursting, termed continued bursting, which occurred in 72.3 ± 2.9% of trials. This raised the question whether this subsequent bursting has an impact on PV. To address this we again collapsed Conditions 1–3 together, given that we found no significant difference between these conditions. We then separated the trials into those with and without continued bursting and compared both groups with regard to their PV (Fig. 4*B*). This confirmed that trials with repeated bursting slow movement down more than those without (*n* = 15, *z* = 15, *p* = 0.008). To disambiguate the effect of re-bursting per se from that of elevation of beta amplitude, we also median split the same burst-triggered trials into groups with low and high mean beta amplitude during the period of continued bursting and compared their PV. The difference was not significant (*n* = 15, *z* = 36, *p* = 0.188; Fig. 4*C*).

### Why might continued bursting impact on peak velocity?

**Figure 5.**
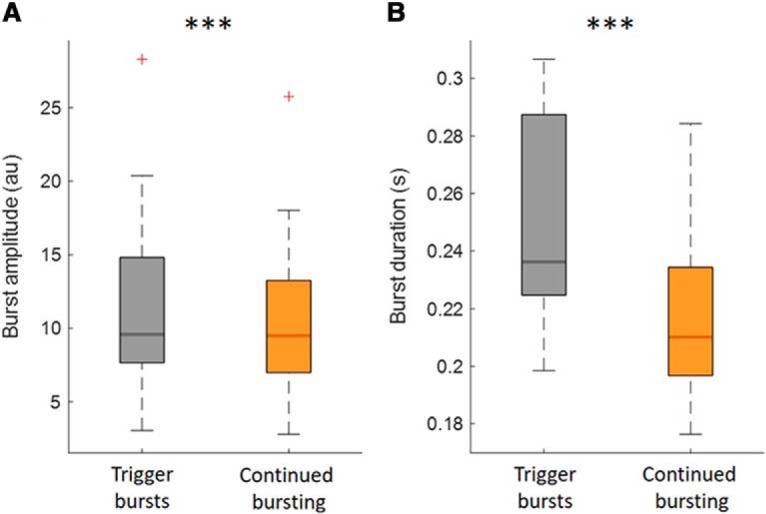
Amplitude and duration of trigger-bursts and of continued bursting. This illustrates that both burst amplitude (***A***) and burst duration (***B***) of trigger-bursts were higher compared with any bursts that followed before movement onset (*n* = 15, *z* = 117, *p* < 0.001; *n* = 15, *z* = 120, *p* < 0.001). Data are averaged across subjects. Red crosses correspond to value above the 75th percentile. ***p < 0.001.

Trials with continued bursting might have had greater impact on PV because subsequent bursts were of longer duration and higher amplitude, given previous reports that suggest that long duration and high amplitude bursts adversely affect motor performance (Tinkhauser et al., 2017a,b; Torrecillos et al., 2018). This simple explanation was explored by comparing the burst characteristics of trigger-bursts and continued-bursting. This showed that continued-bursting was characterized by bursts that were actually lower in amplitude and shorter in duration compared with trigger bursts (Fig. 5).

**Figure 6.**
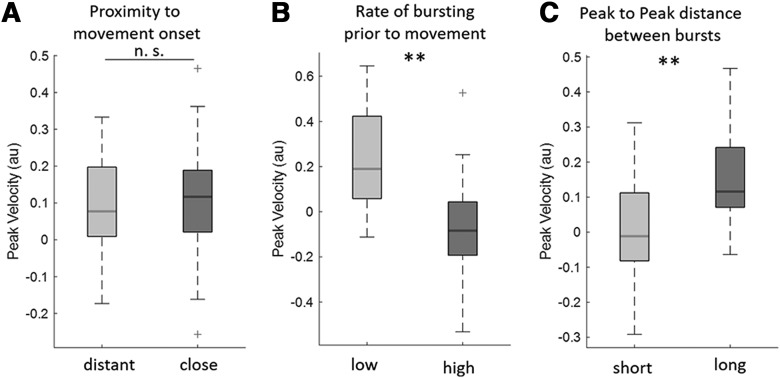
Effect of burst dynamics before movement onset on peak velocity (PV) in Condition 4. Bursting dynamics were studied over the period from −2.5 s to movement onset in the Condition 4 (go-cues not time-locked to bursts). ***A***, Compares two groups (median split) according to whether the amplitude peak of the last burst before movement onset was close to the movement onset (0.29 ± 0.021 s) or further from the movement onset (0.88 ± 0.03 s). No significant difference was found between the two groups (*n* = 15, *z* = 53, *p* = 0.72). ***B***, Compares the trials median split into those with lower (0.84 ± 0.054 bursts/s) and higher rate of bursting (2.09 ± 0.051 bursts/s) before movement onset. Trials with a higher burst rate before movement slowed down more (*n* = 15, *z* = 112, *p* = 0.002). Similar results were reproduced for other time windows (−3 s to movement onset and −2.5 s to movement onset; see main text). ***C***, Compares the effect of interval between bursts before movement onset. Trials are median split into those with shorter (0.41 ± 0.01 s) and longer (0.76 ± 0.02 s) intervals between burst peaks before movement onset. Note, only trials with at least two bursts in the pre-movement period have been included. Trials with bursts occurring at short intervals before movement onset slowed down more (*n* = 15, *z* = 10, *p* = 0.003). Red crosses correspond to value above the 75th or below the 25th percentile. **p < 0.01. n.s. = not significant.

Next, we explored whether continued-bursting was linked to slowing because of the fact that additional bursts are inevitably closer to the movement onset. To this end we focused on Condition 4, in which bursts were just as likely to occur at any time during the 2.5 s period of interest before movement onset (Fig. 2*A*), facilitating segregation into trial subgroups with different characteristics. First, we considered the period from 2.5 s before the movement onset, included all trials with at least one burst and median split these trials according to the proximity of the amplitude peak of the closest burst to the movement onset, resulting in trials where bursts occurred relatively close to movement onset (0.29 ± 0.021 s) and relatively further away from movement onset (0.88 ± 0.033 s). We did not find any significant difference in PV between the two groups (*n* = 15, *z* = 53, *p* = 0.72; Fig. 6*A*). We repeated this procedure for the timing of the end of the last burst instead of the timing of the amplitude peak of the last burst, and this also gave no significant difference (*n* = 15, *z* = 81, *p* = 0.25). Thus, the latency of the last burst with respect to movement onset did not impact on PV within the range of time tested. This result was consistent with the lack of a difference in the effects of Conditions 1–3 on movement slowing.

Second, we considered whether it was the occurrence of multiple bursts in re-bursting that impacted on movement velocity. Accordingly, we applied a median split based on the burst rate (bursts/s) in trials starting from 2.5 s before movement onset. This revealed that trials with a higher burst rate (2.09 ± 0.051 bursts/s) reduced PV more than trials with a lower burst rate (0.84 ± 0.054 bursts/s; *n* = 15, *z* = 112, *p* = 0.002; Fig. 6*B*). This suggests that the occurrence of multiple bursts may have a cumulative negative impact on motor performance. We corroborated this finding by investigating a related measure; whether the time interval between bursts impacted on PV. To this end we did an additional analysis where we only considered trials with at least two bursts before movement onset and median split these according to their burst peak to peak interval. This showed that smaller intervals between the peaks (0.41 ± 0.01 s) of successive bursts were associated with slower PV than larger intervals (0.76 ± 0.02 s; *n* = 15, *z* = 10, *p* = 0.003; Fig. 6*C*). This set of analyses was repeated for periods considering −3 s to movement onset and −2 s to movement onset and showed similar results (burst proximity to movement onset, −3 s: *n* = 15, *z* = 59, *p* = 0.98, −2 s: *n* = 15, *z* = 54, *p* = 0.76; burst rate −3 s: *n* = 15, *z* = 115, *p* < 0.001, −2 s: *n* = 15, *z* = 99, *p* = 0.03; burst interval −3 s: *n* = 15, *z* = 6, *p* < 0.001, −2 s: *n* = 15, *z* = 24, *p* = 0.04). Thus multiple bursts at brief intervals are more relevant for slowing than the simple proximity of the last burst to movement onset.

### Interregional coupling of bursts

**Figure 7.**
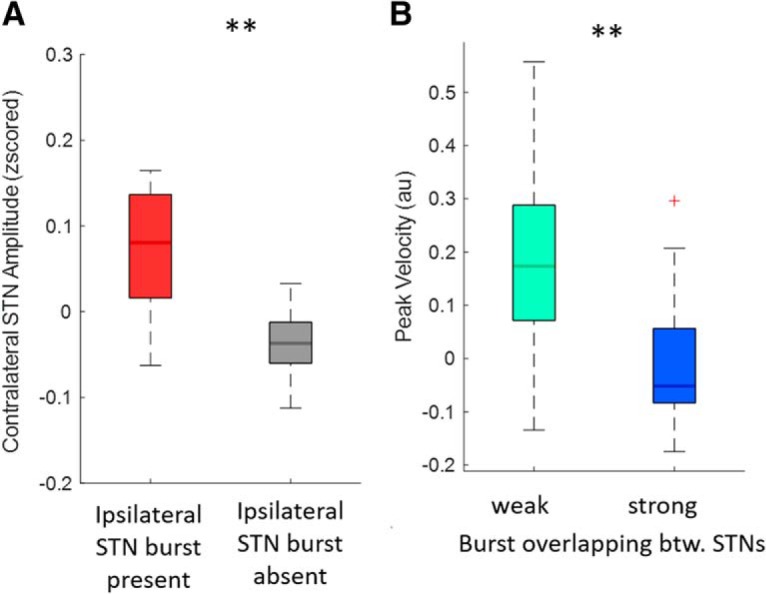
Inter-regional beta burst coupling and slowing of movements. Here the period from −2.5 s to movement onset of reference Condition 4 (go-cues not time-locked to bursts) has been considered. ***A***, Compares the mean amplitude in the cSTN during periods of ipsilateral STN beta bursts and non-bursting periods. The beta amplitude in the cSTN was higher during iSTN beta burst periods compared with iSTN non-burst periods (cSTN, *n* = 15, *z* = 107, *p* = 0.005). ***B***, Compares groups of trials with a stronger degree of burst overlapping (14.8 ± 0.78%time) and weaker burst overlapping (13.49 ± 0.52%time) across hemispheres. This shows that a higher degree of burst overlapping is associated with greater slowing of the movement (*n* = 15, *z* = 112, *p* = 0.001). Red crosses correspond to value above the 75th or below the 25th percentile. **p < 0.01.

Beta bursts have been reported to be coupled across hemispheres (Tinkhauser et al., 2017a,b) and here we explored whether increased long range coupling during beta bursts is also associated with an increased decrement in PV. We again focused on Condition 4 for the same reasons as above and began by confirming amplitude co-modulation across hemispheres during STN beta bursts. We considered the period from 2.5 s before to movement onset and derived burst and non-burst periods in the STN contralateral (cSTN) to the STN responsible for the index bursts (iSTN). For those two periods we compared beta amplitudes in the cSTN. The beta amplitude in the cSTN was higher during iSTN beta burst periods compared with iSTN non-burst periods (Fig. 7*A*). We then determined burst overlapping between iSTN and cSTN. We median split trials according to the percentage time of overlapping of beta bursts between the two STN. This gave one group with weaker (13.49 ± 0.52) and one with stronger (14.8 ± 0.78) percentage overlapping. We then compared the PV of the two groups (Fig. 7*B*). This revealed a significantly lower peak velocity in the group with stronger overlapping (*n* = 15, *z* = 112, *p* = 0.001).

## Discussion

Our results show that the peak velocity of voluntary movements made in response to cues prospectively triggered by STN beta bursts is reduced compared with responses made to cues that are not time-locked to beta bursts. This strengthens the link between beta bursts and slowing of voluntary movements in patients with PD and supports the rationale behind beta amplitude-triggered closed-loop DBS (Little et al., 2013). However, variation in the precise timing of beta bursts within the window before movement onset had no major impact on the decrement in movement velocity, suggesting that the effect of bursts lasted on the order of a second (e.g., the difference between timing of bursts in Condition 3 and motor onset). Such prolonged effects raise the possibility of a cumulative effect of multiple bursts at higher frequency. Examining which features were associated most strongly with slowing we found that multiple bursts within the same trial did indeed seem to be critical. These multiple bursts had to be separated by relatively small intervals and to occur at high rate to be linked to slowing. Moreover, our results suggest that the overlap of bursts between the two STNs was an additional factor for slowing ballistic movements. In sum, these findings suggest that it may be the cumulative, but recent, history of beta bursting in both local and distributed basal ganglia networks that impacts on motor performance in PD.

### Multiple bursts at short intervals impact on motor behavior

It has been shown that beta bursts in untreated PD tend to be prolonged in duration and the proportion of long duration beta bursts is correlated with rigidity and bradykinesia (Tinkhauser et al., 2017a,b; Deffains et al., 2018). More recently it was demonstrated that the occurrence of beta bursts is linked to slowing even at the trial by trial level (Torrecillos et al., 2018). In this study we investigated whether small differences in the timing of bursts before movement had an effect. This was not the case arguing that the functional effects of beta bursts may have a long time-constant, so that the small (∼300 ms) differences in timing between bursts in Conditions 1 and 3 changed the slowing of PV little. This interpretation was supported by the lack of an effect of the delay between the onset and offset of the last burst before movement on movement velocity.

Strikingly, however, if in Conditions 1–3 further bursts occurred after the triggering burst, but before the movement onset, then PV was slowed more than in trials in the same conditions without continued bursting. This suggests that consecutive episodes of bursting might matter. Motivated by this finding, we examined the consequences of episodes of continued bursting observed in Condition 4 in which go-cues were not time-locked to bursts. Here we identified two related aspects of multiple bursting that led to slowing of movements; the rate of bursting, i.e., the number of bursts that occur within a given time window, and the interval between multiple bursts. In contrast, the proximity of the closest burst to movement onset did not affect movement speed over the trial durations analyzed here. Together, our data suggest that multiple bursts occurring at short intervals have a negative impact on motor performance. Parallel findings have been reported in the intact sensory system, where an increased rate of cortical beta bursting impairs sensory processing across species (Shin et al., 2017).

### Long-range synchronization impacts on motor performance

We have previously demonstrated that beta bursts are not simply local episodes of elevated synchrony but also denote episodes of long range, bilateral synchronization in terms of amplitude correlation and phase synchrony across the basal ganglia-cortical motor circuit (Tinkhauser et al., 2018b). Accordingly, we investigated whether episodes of simultaneously elevated synchronization in the two STN would have a greater negative impact on the motor system than unilateral bursts. We showed that trials with prominent burst overlapping between the two STNs led to greater slowing of movements than bursts with little overlapping. Note, though that the simultaneous increase in STN LFP amplitude in both STNs may reflect a neural entrainment originating upstream to the STN, given there is little evidence of lateral connectivity within the STN (Carpenter and Strominger, 1967; Carpenter et al., 1981). Thus, temporal coupling across the motor network enhances the negative impact of bursting on motor performance.

These new observations about the motoric impact of the cumulative, but recent, history of beta bursting across local and distributed basal ganglia networks extend previous findings over longer burst detection periods (spanning minutes instead of seconds) that suggest a correlation between the incidence of beta bursts, particularly those bursts that are more sustained, and bradykinesia and rigidity in patients with PD, as estimated by the motor UPDRS (Tinkhauser et al., 2017a,b). They also extend trial-based analyses which show that both occurrence of a single burst during a critical time window preceding movement and the percentage time spent in bursting during repetitive movements negatively impact motor performance (Torrecillos et al., 2018; Lofredi et al., 2019). These latter effects were not simply explained by mean levels of beta activity, as was also the case here with respect to continued bursting. Complementing these correlative findings is evidence suggesting a causal relationship between beta bursts of longer duration and motor impairment stemming from the observation that terminating such bursts using closed-loop DBS leads to better clinical improvement than randomly delivered stimulation (Little et al., 2013).

### Potential mechanisms whereby beta bursts may impact motor function

Given that episodic increases in beta power in the LFP and EEG index episodes of increased local and intersite synchronization it has been speculated that such episodes might modulate motor function by limiting, at a given moment, information coding capacity within the basal ganglia-cortical system (Mallet et al., 2008; Brittain and Brown, 2014). If so then the functional consequences of temporarily constrained processing may outlive the duration of beta bursts. Indeed, the behavioral effects of beta bursts may outlast bursts by several hundreds of milliseconds whether recorded in health or in PD (Gilbertson et al., 2005; Androulidakis et al., 2008; Shin et al., 2017; Herz et al., 2018; Torrecillos et al., 2018). Short-term plasticity may also contribute to the relatively slow washout of the effects of episodes of elevated beta (Zanos et al., 2018). The slow washout of effects may underpin the cumulative effects of bursting reported here.

### Study limitations and conclusion

The nature of our reference Condition 4 requires further comment. This only contained trials in which the go-cue was triggered randomly with respect to the presence and timing of any beta bursts in the burst screening period. Although the bulk of trials in Condition 4 involved at least one burst in the burst screening period, this was not true of all trials. In some there was no rise in beta that exceeded the threshold for 100 ms or more during the screening period. In other trials the required burst free period of 200 ms in Condition 3 was not met as bursts occurred too frequently and so these trials were classified as belonging to Condition 4. Finally, there were trials in which beta exceeded the threshold but did not then return below this threshold before the end of the burst screening period. However, go-cues were still presented without any systematic locking to beta bursts even given these additional trial types. Moreover, Figure 2*A* shows that the averaged beta amplitude of Condition 4 was similar to that of Conditions 1–3 from 2.5 to 1.5 s before movement onset, but remained flat thereafter. Thus, there was no evidence for an offset in Condition 4 at baseline. The same figure provides good evidence that go-cues were systematically time-locked to beta bursts in Conditions 1–3 but not in our reference Condition 4.

On a more general note, our observations were made in patients in whom recently implanted electrodes had been temporarily externalized. Under these circumstances beta levels may be reduced due to a postoperative stun effect (Chen et al., 2006), and it is not known whether beta dynamics might be similarly affected. We should also acknowledge that clinical evidence of targeting of the STN, and information about localization from the distribution of beta power and its reactivity, is presumptive. Note that data were collected in three different centers, thus implantation techniques and postoperative management of patients might differ slightly. Additionally, we should stress that, with the exception of some evidence from closed-loop DBS (Little et al., 2013), the link between beta bursts, their recent history, and the slowing of movement velocity is correlative. Finally, as our data were collected in parkinsonian patients withdrawn from their medication the inferences made here relate to the link between beta bursts and the reduction of movement velocity in PD, although related findings have been reported in healthy animals and humans (Shin et al., 2017).

Despite these caveats our findings are important in suggesting that it is the cumulative, but recent, history of beta bursting in both local and distributed basal ganglia networks that is linked to slowed movement in patients with Parkinson's disease withdrawn from medication. Treatment with the dopamine prodrug, levodopa, is known to reduce the probability of beta bursts, and this may contribute to its beneficial effects on movement (Tinkhauser et al., 2017b). The present findings also reinforce the argument that beta-amplitude dependent closed-loop DBS should be rapidly reactive, so as to respond to beta bursting (Tinkhauser et al., 2017a).
